# Novel Bioactive and Therapeutic Root Canal Sealers with Antibacterial and Remineralization Properties

**DOI:** 10.3390/ma13051096

**Published:** 2020-03-01

**Authors:** Bashayer H. Baras, Mary Anne S. Melo, Vivek Thumbigere-Math, Franklin R. Tay, Ashraf F. Fouad, Thomas W. Oates, Michael D. Weir, Lei Cheng, Hockin H. K. Xu

**Affiliations:** 1 Department of Advanced Oral Sciences and Therapeutics, University of Maryland School of Dentistry, Baltimore, MD 21201, USA; bbaras@umaryland.edu (B.H.B.); vthumbigere@umaryland.edu (V.T.-M.); toates@umaryland.edu (T.W.O.); 2 Department of Restorative Dental Science, College of Dentistry, King Saud University, Riyadh 11451, Saudi Arabia; 3 Division of Operative Dentistry, Department of General Dentistry, University of Maryland School of Dentistry, Baltimore, MD 21201, USA; mmelo@umaryland.edu; 4 Department of Endodontics, Dental College of Georgia, Augusta University, Augusta, GA 30912, USA; tayfranklin7@gmail.com; 5 Division of Comprehensive Oral Health, Adams School of Dentistry, University of North Carolina, Chapel Hill, NC 27599-7450, USA; afouad@unc.edu; 6 Department of Operative Dentistry and Endodontics, West China School of Stomatology, State Key Laboratory of Oral Diseases, Sichuan University, Chengdu 610000, China; 7 Center for Stem Cell Biology & Regenerative Medicine, University of Maryland School of Medicine, Baltimore, MD 21201, USA; 8 Marlene and Stewart Greenebaum Cancer Center, University of Maryland School of Medicine, Baltimore, MD 21201, USA

**Keywords:** root canal sealer, quaternary ammonium compounds, amorphous calcium phosphate, dimethylaminododecyl methacrylate, antibacterial, remineralization

## Abstract

According to the American Dental Association Survey of Dental Services Rendered (published in 2007), 15 million root canal treatment procedures are performed annually. Endodontic therapy relies mainly on biomechanical preparation, chemical irrigation and intracanal medicaments which play an important role in eliminating bacteria in the root canal. Furthermore, adequate obturation is essential to confine any residual bacteria within the root canal and deprive them of nutrients. However, numerous studies have shown that complete elimination of bacteria is not achieved due to the complex anatomy of the root canal system. There are several conventional antibiotic materials available in the market for endodontic use. However, the majority of these antibiotics and antiseptics provide short-term antibacterial effects, and they impose a risk of developing antibacterial resistance. The root canal is a dynamic environment, and antibacterial and antibiofilm materials with long-term effects and nonspecific mechanisms of action are highly desirable in such environments. In addition, the application of acidic solutions to the root canal wall can alter the dentin structure, resulting in a weaker and more brittle dentin. Root canal sealers with bioactive properties come in direct contact with the dentin wall and can play a positive role in bacterial elimination and strengthening of the root structure. The new generation of nanostructured, bioactive, antibacterial and remineralizing additives into polymeric resin-based root canal sealers are discussed in this review. The effects of these novel bioactive additives on the physical and sealing properties, as well as their biocompatibility, are all important factors that are presented in this article.

## 1. Introduction

There are more than 700 bacterial species that colonize the oral cavity [1,2]. Cracks, caries or trauma to the dental hard tissues provide pathways of entry for these microorganisms to invade the dentinal tubules and reach the pulp tissues [2,3]. However, only a limited number of these microorganisms can colonize and proliferate in the infected root canals [2,4,5]. This is mainly due to the unique and selective nature of the root canal environment that filters out microorganisms that are incapable of surviving the destructive chemicals such as, oxygen radicals, lysosomal enzymes, and nitric acid produced by the host’s immune system [2,5]. 

Microorganisms within the root canal system are organized in a complex biofilm structure composed of an extracellular polymeric substance (EPS) matrix derived from substances produced by the host or the microorganisms themselves [6,7,8]. In addition, polysaccharides, proteins, lipids, and nucleic acids produced by microorganisms further enhance the formation and complexity of the biofilm structure [2,7]. Microorganisms organized in a biofilm structure have shown to be more than one thousand times more resistant to antimicrobial agents than their planktonic counterparts [2]. 

It was estimated that in 2007, approximately 15.7 million root canal treatments and retreatments were performed annually, with an average of 41,000 root canal treatments performed each day in the United States [9]. Root canal therapy aims to completely eliminate microorganisms or allow for their maximal reduction to allow the host immune system to heal and regenerate the damaged peri-radicular tissues [5,10,11]. Various methods have been employed to disinfect the root canal space, which include mechanical instrumentation, chemical irrigation, applications of intracanal medicaments to further enhance microbial elimination, and finally the obturation of the root canal space with an inert and biocompatible material [2]. 

The main objectives of the obturation process are as follows: (1) prevent the ingress of oral fluids; (2) entrap residual surviving bacteria and deprive them from nutrients and; (3) prevent any communications between periapical tissue fluids and the root canal, all of which can act as a source of future infections [12]. However, despite major efforts to disinfect the root canal system, due to its complex anatomy and resistant nature of residing biofilms, failure of root canal therapy still occurs. 

Previous review articles on root canal sealers have mainly focused on commercially-available root canal sealers and their properties [13,14]. A recent review article mainly focused on the bioactive modification of epoxy-resin based root canal sealers through the addition of antimicrobial agents [15]. The present review focuses on recent advances made to modify resin-based root canal sealers, including both epoxy and methacrylate-resin based root canal sealers, with novel bioactive antimicrobial and remineralizing additives. Agents such as silver, chitosan, quaternary ammoniums, and calcium phosphate nanoparticles are the focus of this review. The effects of combining multiple bioactive agents for synergistic and maximal benefits, and the influence on physical and biological properties are discussed in the present review. 

## 2. Resin-Based Root Canal Sealers and Their Properties

Root canal sealers used in combination with solid core materials can provide a fluid-tight hermetic seal [12,13]. They are used mainly to fill in gaps present between the core filling material and the dentinal wall. Gutta-percha (GP) is the most commonly used solid-core filling material, and only few materials were used as alternatives to GP [12]. Researchers have mainly focused on finding an ideal root canal sealer to use in combination with GP. Polymeric resin-based root canal sealers were introduced to overcome shortcomings associated with conventional zinc oxide eugenol (ZOE) sealers [13,16,17,18]. Those shortcomings include inability to strengthen root structure, lack of bonding to dentin, microleakage, and the high solubility, all of which may compromise the longevity of root canal therapy [13,14,15,16]. 

Polymeric root canal sealers, such as silicon-based sealers and resin-based sealers were introduced to the market to overcome limitations associated with conventional sealers. AH Plus (Dentsply DeTrey, Konstanz, Germany) is an epoxy-resin based paste-paste sealer system [13] and is widely accepted in root canal therapy. It consists of an epoxide paste and an amine paste that consists of three different types of amines [13]. AH Plus has demonstrated low solubility and good dimensional stability in solutions. It can adhere to root dentin due to its creep properties and long setting times [13,19,20]. Regarding the antimicrobial properties, epoxy-resin based sealers have shown to produce some antibacterial properties but mainly before their setting due to the release of some of the constituents like formaldehyde [21,22]. Many efforts have been made to prolong the antimicrobial properties of epoxy-resin sealers through the addition of antibacterial agents, such as silver, quaternary ammonium compounds, chlorhexidine, calcium hydroxide and many more, which have shown improved antimicrobial activity and minimal adverse effects on physicochemical and biological properties [15]. 

Methacrylate-resin based root canal sealers were introduced to provide the concept of a “mono-block” by bonding the core filling material to the canal wall and forming a single unit [23]. They were developed to provide a better seal and mechanically reinforce compromised roots, which have been suggested to reduce bacterial ingress pathways and strengthen the root structure [13,14,23]. Methacrylate-based sealers are meant to infiltrate the partially demineralized collagen matrix and create micromechanical retention to root dentin [22,23]. Despite the desirable concept of the mono-block, the lack of relief of polymerization shrinkage stresses associated with these sealers that occur as a result of the unfavorable cavity configuration of the root canal, result in the pulling out of the resin tags from the dentinal tubules [22,23]. This can compromise the sealer-dentin bond and result in micro-gaps that can act as a source for micro-leakage [22,23]. For these specific reasons, efforts have been made to improve the bond and antibacterial properties of methacrylate-resin based root canal sealers [24,25,26,27].

## 3. Resin-Based Root Canal Sealers as Carriers for Bioactive Additives

Modifications to root canal sealers have been made to improve their antimicrobial properties and promote the longevity of root canal therapy. These efforts have mainly focused on the incorporations of soluble bioactive, antibacterial additives like silver, or through the copolymerization of antibacterial agents like Quaternary Ammonium Compounds (QACs) within polymeric materials to produce long lasting effects, which have gained a wide-spread interest [24,25,26,27]. 

Another issue that has gained the attention of many researchers, is the adverse effects exerted by some of the most commonly used root canal irrigants like, sodium hypochlorite (NaOCl), calcium hydroxide paste, ethylenediamine tetra acetic acid (EDTA), and chlorhexidine gluconate solution (CHX) on dentin mineral composition [28,29]. Previous studies have demonstrated the ability of these solutions to cause mechanical and structural damage to radicular dentin, resulting in a brittle dentin structure that is more susceptible to fracture [30,31]. In addition, the acidic nature of some chelating agents like acetic acid and citric acid used during removal of the smear layer may contribute to the dissolution of tooth minerals. Remineralizing fillers were incorporated into root canal filling systems to reverse the action of demineralizing irrigation solutions and increase the hardness of the dentin structure through the deposition of tooth minerals [27,32,33]. The availability of remineralizing ions at high concentrations near the dentin surface favors their deposition and promotes remineralization. 

## 4. Antibacterial Additives into Resin-Based Root Canal Sealers

Antimicrobial agents exert their effects based on one of three different strategies: (1) through their local release; (2) through contact-killing; or (3) through multi-functional strategy to produce synergetic properties [34]. The latter offers the advantage of overcoming the drawbacks associated with the exhaustion of the releasing agents and the short-distance killing of the contact-mediated agents. Antibacterial dental resins can also be classified into (1) polymerizable quaternary ammonium compounds and (2) antibacterial filler particles. In the first classification, the antibacterial agent is polymerized within the resin matrix through covalent bonding and does not leach out from the material [35]. Below are some of the most commonly used antimicrobial agents incorporated separately or in combination into root canal sealing materials (Table 1). 

### 4.1. Releasing Antimicrobial Additives

Back in the 1950s, Colten introduced the idea of releasing antimicrobial agents when he incorporated antibiotics into dental fillings [34,36]. This strategy allows for the local delivery of high concentrations of the preloaded antimicrobial agents without dealing with the adverse toxic effects related to systemic drug delivery [34]. Since then, this strategy has gained wide popularity. However, due to the emerging antibacterial resistance and allergic host response to antibiotics, their frequent use is not recommended [34]. Other releasing antimicrobial agents have been introduced and incorporated into various dental materials. However, one main drawback of incorporating these releasing antimicrobials into dental materials is their initial burst release followed by the depletion of their release over time [37,38]. 

#### 4.1.1. Silver and Silver Nanoparticles

Silver (Ag) is one of the most commonly used antimicrobial agents that produce wide spectrum antimicrobial properties [39,40]. It demonstrated strong antibacterial, antifungal, and antiviral effects when incorporated into various dental resins [38,39,40]. The antibacterial mechanism of action of silver nanoparticles mainly relies on the release of silver ions that target multiple sites on the bacterial cell [41]. For example, silver ions can increase the bacterial wall membrane permeability by physically adhering to it, thus influencing the influx and efflux of essential ions [41]. The adherence of silver ions to the bacterial cell wall can also cause cell wall destruction and release of essential cellular components [41]. Due to the high affinity of silver to sulfur, nitrogen and oxygen, silver ions can bind to sulfhydryl groups in proteins and cause protein denaturation or bind to N atoms from nucleic acids and prevent DNA replication [42].

The antibacterial efficacy of silver particles has shown to be concentration dependent. The ability of the silver particles to release high quantities of silver ions becoming available for interaction with the bacterial cell is what determines their antibacterial potency [38]. In a study by Wang et al., polyurethane (PU)-based sealers with different concentrations (1 wt %, 3 wt %, and 5 wt %) of silver phosphate (Ag_3_PO_4_) particles were formulated [43]. The antibacterial results showed that increasing the concentration of Ag_3_PO_4_ to 3% and 5% demonstrated stronger antimicrobial effects than that achieved with 1% Ag_3_PO_4_ and an epoxy-resin sealer AH Plus control when tested against *Streptococcus mutans (S. mutans)* utilizing an anti-adhesion assay and a direct contact antibacterial test [43]. 

The incorporation of silver nanoparticles into root canal sealers when compared to micro-sized particles provides an additional benefit due to their nanoparticle characteristics and small particle size that allow them to exert potent antimicrobial effects at decreased filler levels. Teixeira et al., incorporated nanostructured silver vanadate decorated with silver nanoparticles (AgVO_3_) into epoxy-resin Sealer 26 and AH Plus sealer in three concentrations: 2.5 wt %, 5 wt % and 10 wt % and investigated the effects of their addition on the minimum inhibitory concentration (MIC) against *Enterococcus faecalis* (*E. faecalis*), *Pseudomonas aeruginosa* (*P. aeruginosa*) and *Escherichia coli* (*E. coli*) [44]. An agar diffusion test was used to evaluate the materials’ antibacterial effects after 48 h and 7 days [44]. The materials’ flow and radiopacity were also evaluated. The MIC results were reported to be 500 µg/mL for *E. faecalis* and 31.25 µg/mL for *P. aeruginosa* and *E. coli* [44]. When silver vandate was incorporated into AH Plus at all three concentrations, no significant effect on the inhibition zones against *E. faecalis* were observed regardless of the incubation time [44]. However, modifying Sealer 26 with silver vandate at concentrations of 5% and 10% enhanced the antibacterial potency [44]. Both AH plus and sealer 26 modified with silver vandate showed no significant increase in the diameter of the inhibition zone against *P. aeruginosa* and *E. coli* [44]. Only 10% of silver vanadate in AH Plus provided zones of inhibition against *P. aeruginosa*. There was no statistical difference in the zones of inhibition between the groups of 48 h and 7 days [44]. 

In a recent study, nanoparticles of silver (NAg) with a diameter of 2.7 nm were synthesized in situ, where a polymer matrix was used as a stabilizing agent [45]. This was achieved by dissolving silver salts that are poorly soluble in methacrylate resins in a methacrylate-based solution to allow the silver salt to be chemically incorporated into the resin matrix upon polymerization [45]. This method was developed to prevent nanoparticle aggregation and agglomeration commonly associated with the direct incorporation of silver nanoparticles into resin matrices [45]. In addition, through the chemical incorporation of the silver salts in the resin matrix this allowed for the entrapment of silver nanoparticles within the matrix while releasing silver ions at a gradual and sustained rate [38,45].

Seung et al. investigated the effects of incorporating dimethylaminododecyl methacrylate (DMAHDM) and NAg into AH Plus on the antibacterial properties utilizing a modified direct contact test at 1, 7, and 14 days against *E. faecalis* [24]. When the NAg was incorporated at 0.15%, a one log CFU reduction was achieved at day 1, but no reduction was observed at days 7 and 14 [24]. One possible explanation could be that the incompatible mixing of the methacrylate-based NAg into the epoxy resin-based AH Plus sealer resulted in the complete depletion of silver at day 1. When another study incorporated DMAHDM and NAg into a methacrylate-resin based experimental sealer, 0.15% NAg was able to achieve a 2 log CFU reduction against *E. faecalis* biofilm grown on saliva-coated resin disks, when compared to experimental control group without NAg [26]. Combining 0.15% NAg and 5% DMAHDM resulted in a 6 log CFU reduction [26]. Sealer with 0.15% NAg was also able to significantly reduce biofilm polysaccharide production [26]. When resin disks with NAg were observed under an epifluorescence microscope after being subjected to live/dead staining, they showed less live bacteria and more dead bacteria when compared to control resin disks without NAg [26]. 

#### 4.1.2. Chlorhexidine

Chlorhexidine is another common antimicrobial agent that has been commonly incorporated into root canal materials. Chlorhexidine when compared to other antimicrobial drugs has a broader antimicrobial spectrum [46]. It is effective against gram negative, gram positive bacteria, and yeast. It has a quick kill rate and is considered to have an instant bactericidal effect and a sustained bacteriostatic action depending on its concentration [46,47]. The mechanism of action of chlorhexidine is through the interaction of the positive charge of the molecule and negatively charged phospholipids and lipopolysaccharides on the microbial cellular membrane [46,47,48]. 

Sanchez and colleagues tested the effects of mixing AH Plus, with chlorhexidine alone or in combination with cetrimide (CTR) against *E. faecalis* biofilm inhibition [49]. The authors incorporated 1% and 2% CHX and 0.1–0.5% CTR alone or in combination into AH Plus sealer and assessed their antimicrobial activity utilizing a modified direct contact test and a 24-hour *E. faecalis* biofilm through the Calgary biofilm device (MBEC-high throughput [HTP]; Innovotech, Edmonton, AB, Canada) [49]. The results of the study showed that when 2% CHX was incorporated into AH Plus a 1.3 log CFU reduction against *E. faecalis* was achieved [49]. None of the used concentrations were able to completely eradicate or inhibit biofilm when compared to unmodified AH Plus sealer [49]. When CHX and CTR were combined together at 2% and 0.5%, respectively, CFU log reduction was increased to 6.28 [49]. 

The results from the afore-mentioned studies provide promising methods to enhance the elimination of microorganisms hidden away in the complex root canal anatomies. Releasing antimicrobials can target microbes away from the canal wall surface that are usually difficult to eliminate through traditional instrumentation and irrigation techniques. However, further studies utilizing more complex biofilm models should be conducted. In addition, the extent of the release of these antimicrobials should be measured to determine their depth of penetration into the dentinal tubules.

### 4.2. Contact-Killing Antimicrobials

#### 4.2.1. Quaternary Ammonium Compounds

Quaternary ammonium compounds have been developed and chemically incorporated into various polymeric resin matrices of dental materials [50,51,52,53,54]. These compounds can produce long term antibacterial effects without leaching out and becoming depleted over time through the formation of cross-links with other compounds [54,55]. The mechanism of action of QACs is identified as contact-inhibition [55]. When the negatively charged bacterial cell contacts the positively charged (N+) sites of QACs, an electrical imbalance occurs in the bacterial cellular membrane and the bacterium bursts under its own osmotic pressure [55]. 

Various chemical compositions have been developed and incorporated into various dental materials. Imazato and colleagues were among the first investigators to develop quaternary ammonium methacrylates which were chemically copolymerized into dental resins yielding potent and long-lasting antibacterial effects [55]. Their bonding agent containing 12-methacryloyloxydodecylpyridinium bromide (MDPB), Clearfil Protect Bond, was the first to become commercially available [56]. It was able to produce potent antibacterial effects against various cariogenic and endodontic species like *Streptococcus mutans, Lactobacillus casei* and *Actinomyces naeslundii* [56]. 

In a previous study, 5% MDPB was incorporated into an experimental methacrylate-based root canal primer (EP) used in combination with a Bis-GMA-based sealer [57]. The study investigated MDPB’s antibacterial effects against planktonic or adherent *E. faecalis* bacteria for 30 or 60 s. The authors also utilized a root canal infection model to determine the antibacterial effects of MDPB against *E. faecalis* impregnated within the dentinal tubules [57]. Their results showed that EP containing MDPB was able to significantly reduce the number of viable planktonic and adherent bacteria when compared to Epiphany, a commercially available sealer at the time and experimental sealer without MDPB. After 30 s contact, a 99.9% killing of planktonic bacteria was achieved and 100% killing was achieved after 60 s [57]. For adherent bacteria, MDPB killed more than 98% in 30 s and 99% in 60 s [57]. A 99.5% killing was achieved by MDPB for bacteria impregnated inside dentinal tubules [57]. 

Dimethylaminododecyl methacrylate (DMADDM) is a QAM with an alkyl chain of 12 that has been previously incorporated into composites and adhesive systems and was able to produce potent antibacterial effects [58,59,60]. In a recent study, DMADDM was incorporated into EndoREZ, a urethane dimethacrylate (UDMA)-based root canal sealer [61]. The authors investigated the antibacterial effects of adding 0%, 1.25%, 2.5%, and 5% of DMADDM on multispecies bacteria (*E. faecalis, Streptococcus gordonii, Actinomyces naeslundii, and Lactobacillus acidophilus*), in planktonic cells as well as biofilms [61]. When DMADDM was added at 1.25% and 2.5%, there was a significant bacterial reduction of planktonic bacteria even after the sealers were set for 10 days [61]. The authors also reported that the eluents from the sealers containing DMADDM did not produce significant antibacterial effects, which emphasized the contact-killing mechanism and the cross-linking of DMADDM with EndoREZ [61]. When assessing the multispecies biofilm-inhibition by DMADDM, EndoREZ containing 2.5% DMADDM achieved a CFU reduction of 1 order of magnitude when compared to groups with 0% DMADDM, as well as significant biofilm biomass reduction [61]. Furthermore, DMADDM reduced the proportion of *E. faecalis* in the biofilm from 31% to 23% [61].

**Figure 1:**
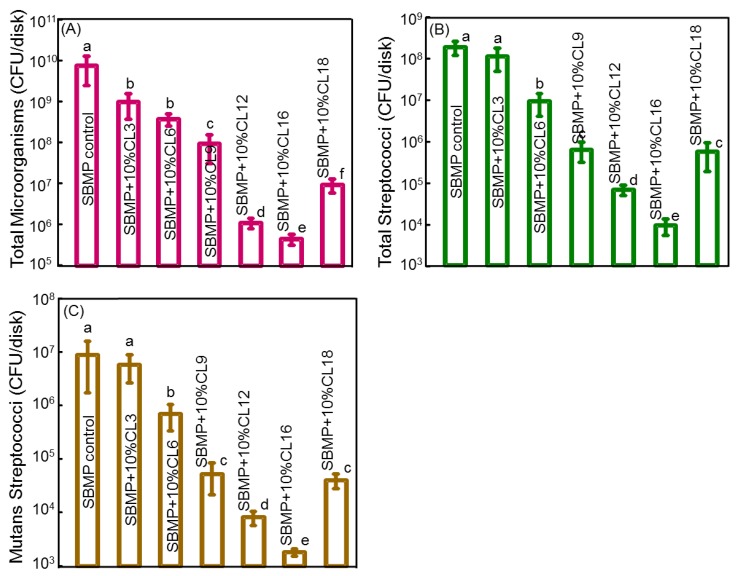
Effect of chain length (CL) on dental plaque microcosm biofilm colony-forming units (CFU): (**A**) Total microorganisms, (**B**) total streptococci, and (**C**) mutans streptococci. Two-day biofilms on resins were used for CFU measurements (mean ± sd; *n* = 6). Note the log scale for the *y*-axis. The CFU at CL of 16 was 4 log lower than that of SBMP control. In each plot, values with dissimilar letters are significantly different from each other (*p* < 0.05). Adapted from [50] with permission.

To better enhance the antibacterial effects of QAMs, efforts have been made to increase their alkyl chain length. Recently, QAMs with various alkyl chain lengths, including 3, 6, 12, 16, and 18, were synthesized and incorporated into a dental bonding system [50]. Increasing the alkyl chain length significantly increased the antimicrobial activity [50]. The authors proposed that QAMs with longer alkyl chains have dual antibacterial mechanisms: (1) The electrical imbalance due to their positive charge and (2) the ability of their long chains to physically pierce bacterial membranes [50]. Dimethylaminohexadecyl methacrylate (DMAHDM) with chain length 16 showed the strongest antibacterial activity [50] (Figure 1). Recently, 2.5% DMAHDM was incorporated into AH Plus sealer in combination with 0.15% NAg [24]. The authors utilized the DCT to assess the antibacterial properties against planktonic *E. faecalis* over 14 days [24]. Groups with DMAHDM maintained a significant CFU reduction over the 14 day period [24]. Another study incorporated DMAHDM into a Bis-GMA/ TEGDMA based root canal sealer to overcome drawbacks associated with the incompatible mixing of DMAHDM (methacrylate resin-based), and AH Plus (epoxy-resin based) and to better asses the antibacterial effects of DMAHDM against bacteria in biofilms [62]. The authors investigated the antibacterial effects associated with the addition of 5% DMAHDM into the experimental sealer on *E. faecalis* biofilm grown on saliva-coated resin sealer disks and compared the results with AH Plus sealer [62]. The authors reported a 4 log CFU reduction and significant reduction in biofilm polysaccharide production by *E. faecalis* [62]. When sealer disks were subjected to live/dead staining, sealers containing 5% DMAHDM were primarily covered with bacteria with compromised membranes unlike AH Plus sealer disks which were covered mainly with live bacteria [62].

**Figure 2:**
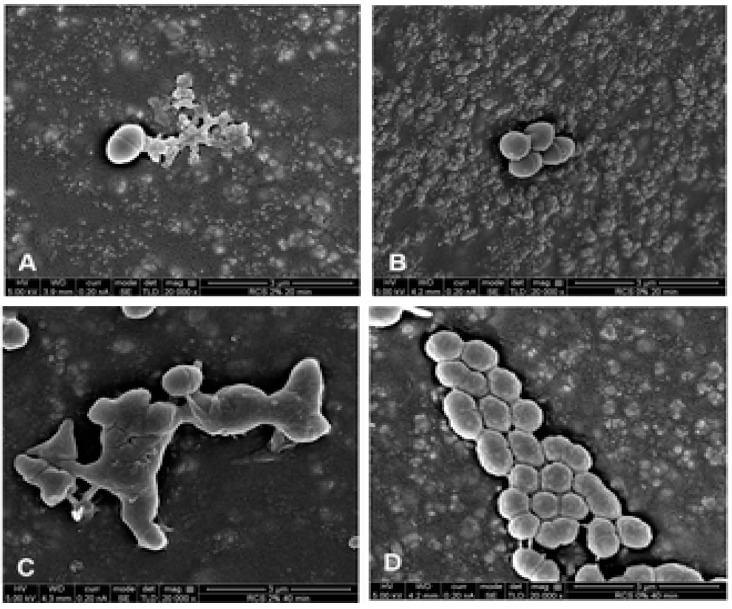
Images of *E. faecalis* following contact with the novel endodontic sealer and unmodified sealer. (**A**) *E. faecalis* following 20 min direct contact with the surface of the novel endodontic sealer. Syncytium like cells and bacterial lysis can be observed. Most of the bacteria show changes in morphology with no visible signs of cell division. (**B**) Bacteria following 20 min direct contact with a sealing material without QPEI nanoparticles, early biofilm formation with intact membrane and dividing cells can be observed. Bacteria on the surface of the novel sealer and the sealer without nanoparticles after 40 min, (**C**) and (**D**) respectively. Adapted from [66].

Quaternary ammonium polyethyleneimine (QPEI) have shown to produce potent antibacterial properties [63]. These antibacterial quaternary ammonium-based nanoparticles were previously incorporated into various dental resins that were able to inhibit biofilm formation both in vitro and in vivo [64,65]. Beyth et al. used a polyethyleneimine scaffold to synthesize quaternary ammonium-based nano-sized particles (QPEI) that were incorporated into an epoxy-based endodontic sealer “RCS” [66]. The authors were able to affix the nanoparticles within the sealer to prevent their release and depletion over time [66]. When tested against *E. faecalis*, incorporating 1.5% QPEI nanoparticles significantly reduced the number of residual viable bacteria by 6 logs when compared to the unmodified RCS sealer [66]. SEM analysis of the QPEI containing sealer revealed significant changes in bacterial morphology and distribution (Figure 2). Syncytium-like cells and bacterial lysis were observed on sealers containing QPEI nanoparticles [66]. 

#### 4.2.2. Chitosan

Another cationic antibacterial agent that has been commonly incorporated into various dental materials is chitosan. The mode of action of chitosan is also linked to the electrical interaction between the positive charge of NH_3_^+^ groups of the polycationic chitosan and the negative charge of the bacterial cellular membrane resulting in the destruction of the bacterial cellular wall [67]. In a recent study, chitosan nanoparticles (CNps) were incorporated into Epoxy resin- and calcium silicate–based sealers, ThermaSeal and MTA Fillapex, respectively [67].

CNps were added to the sealers at 1 g/150 mg [67]. The authors investigated the antibacterial effects of the modified sealers utilizing the direct-contact and membrane-restricted antibacterial experiments [67]. The authors reported significant improvement in the antibacterial properties of Thermseal and MTA Fillapex against *E. faecalis* after the incorporation of CNps [67]. The authors also assessed the antibiofilm effects of chitosan using a root canal infection model, where *E. faecalis* biofilm formation was assessed at the sealer dentin interface of roots treated with carboxymethyl chitosan (CMCS) or CMCS+rose bengal and filled with gutta-percha and CNps modified sealers [67]. When samples were viewed under confocal laser scanning microscopy a high viable biofilm total volume at the sealer–dentine interface was observed after 4 weeks in the unmodified sealers without CNps [67]. Samples filled with sealers containing CNps showed significantly lower values and much less bacterial colonization [67].

#### 4.2.3. Cetrimide

Cetrimide is another cationic compound that has demonstrated strong antibacterial activity. When used as a root canal irrigant, it was able to eradicate *E. faecalis* biofilms at concentrations as low as 0.0078% in approximately 2 mins [68]. In a study, the effects of mixing AH Plus with chlorhexidine (CHX) and cetrimide (CTR) alone or in combination against *E. faecalis* biofilm formation were assessed [49]. The authors assessed the antibacterial effects of CTR at 0.1–0.5% utilizing a direct contact test against *E. faecalis* in biofilm for 24 h [49]. The effect of CTR seemed to be concentration dependent [49]. A log10 reduction of 2.92 at 0.5% and inhibition of biofilm formation at 0.2% was achieved in groups containing CTR [49]. Lower concentrations of CTR and CHX combined together were needed to achieve similar results [49]. 

The incorporation of antimicrobial agents with contact-killing properties like MDPB and DMAHDM could potentially prevent secondary root canal infections in cases of microleakage and bacterial proliferation. This is because in the case of microleakage and bacterial invasion, the bacteria would come into contact with the sealer resin at the interface which would exert contact-killing effect to kill the bacteria. Therefore, these contact-killing materials could potentially extend the longevity of root canal treatment and minimize the need for future re-treatments. 

## 5. Remineralizing Additives into Polymeric Root Canal Sealers

In addition to having antimicrobial properties, an ideal root canal sealer should induce the formation of mineralized tissue. Calcium and phosphate ions are essential for the formation of hydroxyapatite [Ca_10_(PO_4_)_6_(OH)_2_], the major mineral component of teeth [38]. Therefore, it would be beneficial to develop a root canal sealer that could release calcium and phosphate ions to form hydroxyapatite. The development of a remineralizing root canal sealer could reverse the action of root canal irrigants and chelating solutions such as NaOCl and EDTA. They could alter the chemical structure of dentin, resulting in damage, mineral loss and reduction in root dentin hardness, thus leading to tooth fracture [31,69]. 

Several classes of calcium-phosphate and calcium silicate root canal sealers have been developed. Their major benefits are to produce high pH and calcium and phosphate ion release to promote mineralized tissue formation [70]. Recently, Inorganic fillers were incorporated into polymeric resin matrices and tested for their potential to release high levels of Ca and P ions and strengthen the tooth root structure [54]. Fillers such as dicalcium phosphate dihydrate (DCPD), dicalcium phosphate anhydrous (DCPA) and tetracalcium phosphate (TTCP), alpha tricalcium phosphate (α-TCP) have previously shown their potential to release high levels of calcium and phosphate ions and remineralize enamel and dentin lesions [32,71,72,73]. 

Alpha-tricalcium phosphate is a soluble compound with a solubility product of 3.16 × 10^−26^ and a Ca/P ratio of 1.5 [74]. It can interact with human tissue fluids to release supersaturated levels of Ca and P ions that stimulate mineral deposition on the sealer-root interface promoting remineralization and healing of periapical tissues [32]. Poretlla et al. tested the effects of incorporating α-TCP in 0, 5, 10, 15 % wt in combination with calcium hydroxide into a 70% glycerol salicylate resin to be used as an endodontic sealer [33]. The authors subjected their α-TCP containing sealers to Raman spectroscopy and SEM-EDX immediately after hardening and after storage in distillated water or simulated body fluid (SBF) for 7 days for surface characterization of the samples and determination of their bioactivity [33]. All sealers showed similar surface morphology before immersion [33]. However, SEM images revealed that sealers containing α-TCP presented irregular surfaces that were needle-like with calcium and phosphorous deposition [33]. The EDX analysis after water immersion revealed an increase in calcium and phosphorus on the surface of the specimens containing α-TCP [33].

Rostirolla et al. investigated the effects of incorporating 10 wt % of hydroxyapatite (HAp), α-TCP, or octacalcium phosphate (OCP) particles into an experimental methacrylate-resin based endodontic sealer on mineral deposition [32]. The mean particle size for HAp, α-TCP, OCP particles were 26.8 nm, 4.94 μm, and 6.03 μm, respectively [32]. Micro-Raman interface and mineral deposition analysis were performed. Groups containing HAp presented the highest mineral deposition on the specimen’s surface [32]. The authors explained that HAp particles exhibit higher thermos-dynamic stability and lower solubility among the other tested calcium phosphates, which explains their higher mineral deposition [32]. In addition, the highest percentage of calcium ion release was achieved in groups with HAp from 7 to 28 days (14.13%−40.86%, respectively) [32]. Groups containing OCP showed no apatite deposition, mainly attributed to their high solubility, which could lead to the precipitation of less stable mineral particles that can be easily washed out during immersion [32].

Amorphous calcium phosphate (ACP) particles have been previously incorporated into various dental resins [53,58,75,76]. ACP is the initial phase that forms and then transfers into the more stable Hydroxyapatite [HA: Ca_10_(PO_4_)_6_(OH)_2_], through the precipitation of calcium and phosphate ions in supersaturated levels [54]. In fact, previous studies have shown the ability of ACP containing materials to release high levels of Ca and PO_4_ ions in aqueous environments, necessary to remineralize the enamel lesions and dentin lesions [77]. 

**Figure 3:**
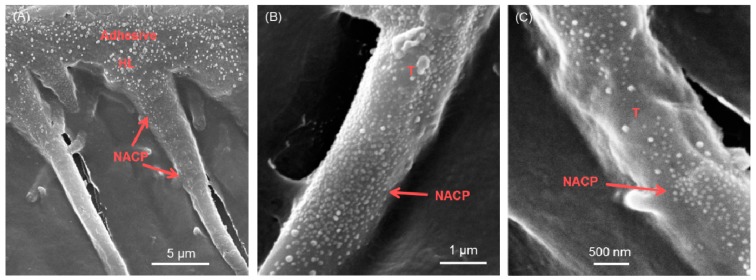
NACP in dentinal tubules. (**A**) Representative scanning electron microscopy (SEM) micrograph showing numerous NACP within the resin tags. (**B**,**C**) SEM micrographs of NACP particles within the resin tags at higher magnifications. Adapted from [78] with permission.

One drawback related to the incorporation of micro-sized ACP particles is the poor mechanical properties they exert on the material. Recently, through the application of nanotechnology, nanoparticles of amorphous calcium phosphate (NACP) were synthesized with a mean particle size of 112nm. Due to their higher surface area and greater Ca and P ion release than their micro-sized counter parts, NACP were able to remineralize enamel lesions and dentin lesions in vitro while maintaining the necessary mechanical and bonding properties of the material [51,52]. Previous TEM images have shown the ability of NACP to flow with dental resins into dentinal tubules while releasing high levels of calcium and phosphate ions [78] (Figure 3). 

**Figure 4:**
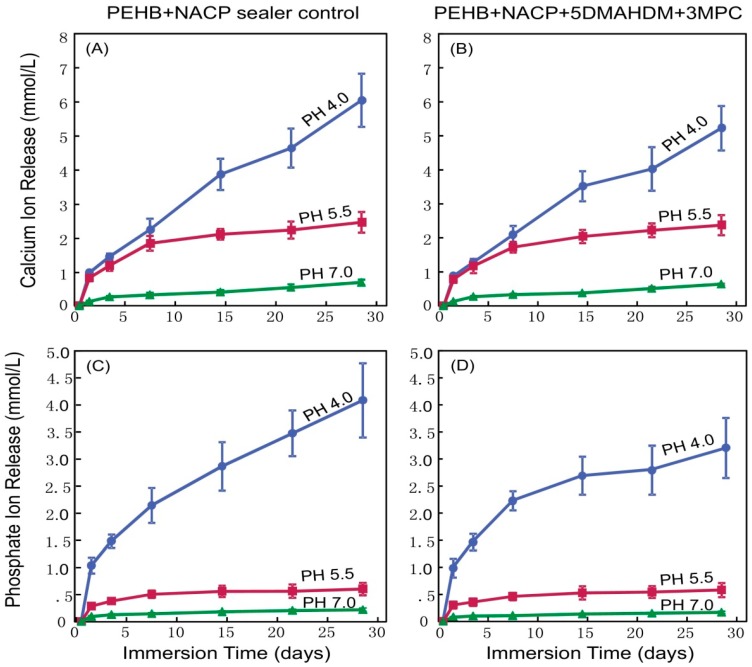
Calcium (Ca) and phosphate (P) ion release from endodontic sealers PEHB + NACP control and PEHB + NACP + 5DMAHDM + 3MPC. (**A**,**B**) Ca ion release, (**C**,**D**) P ion release. Ca and P ions concentrations increased with increasing time and decreasing pH. Adapted from [79] with permission.

In a recent study, 20% NACP was incorporated into an experimental methacrylate-resin based antibacterial root canal sealer containing DMAHDN and MPC [79]. The authors investigated the Ca and P ions release when samples were immersed in a sodium chloride solution buffered at three different pH levels (4, 5, and 7) [79]. NACP containing sealer was able to release high levels of Ca and P ions reaching approximately 6 and 4 mmol/L, respectively at pH 4 [79]. The ion concentrations increased with time and decreasing pH (Figure 4) [79]. The smart release of NACP at higher levels in decreasing pH levels is especially beneficial because these ions are usually needed in acidic conditions when demineralization of the tooth structures occurs. In addition, NACP-containing dental resins have shown their ability to raise the pH of acidic solutions that usually favor the growth of anaerobic bacteria [27]. In a recent study, NACP particles were incorporated into an experimental methacrylate resin-based root canal sealer at three mass fractions of 10%, 20%, and 30% [27]. Sealers with 30% NACP were able to raise a sodium chloride solution pH from 5 to 6.32 in approximately 60 mins [27]. In addition, when the authors treated dentin with 5.25% NaOCl and 17% EDTA, the dentin microhardness was significantly reduced [27].

However, treating dentin specimens with a sealer containing 30% NACP significantly increased the dentin hardness from 0.37 GPa to 0.52 GPa, which was not significantly different from that of sound dentin [27]. It is well-known that microhardness analysis provides an indirect indication of mineral loss or gain, thus an increase in dentin hardness indicates an increase in the deposition of Ca and P ions. 

## 6. Combining Bioactive Antimicrobial and Remineralizing Agents into Root Canal Sealers for Multifunctional Purposes

Developing a bioactive root canal sealer with multifunctional antibacterial and remineralizing agents that complement each other is highly desirable. Releasing agents such as, particles of NAg can be released into complex areas of the root canal system that are usually difficult to access through traditional instrumentation and irrigation techniques [26]. In fact, *E. faecalis*, a common bacterium isolated from previously treated root canal treated cases, with or without apical periodontitis, can invade the dentinal tubules to a depth of 200 to 1500 μm, with greater depth of penetration and number of infected tubules in dentin from young patient [80,81]. In addition, antibacterial agents with contact killing properties, like QACs can kill bacteria that come in contact with the material in cases of microleakage, which could prevent future re-infection [26,27]. Remineralizing fillers can also be incorporated to remineralize tooth lesions and strengthen the tooth root structures, preventing future tooth fractures and subsequent tooth loss. 

**Figure 5:**
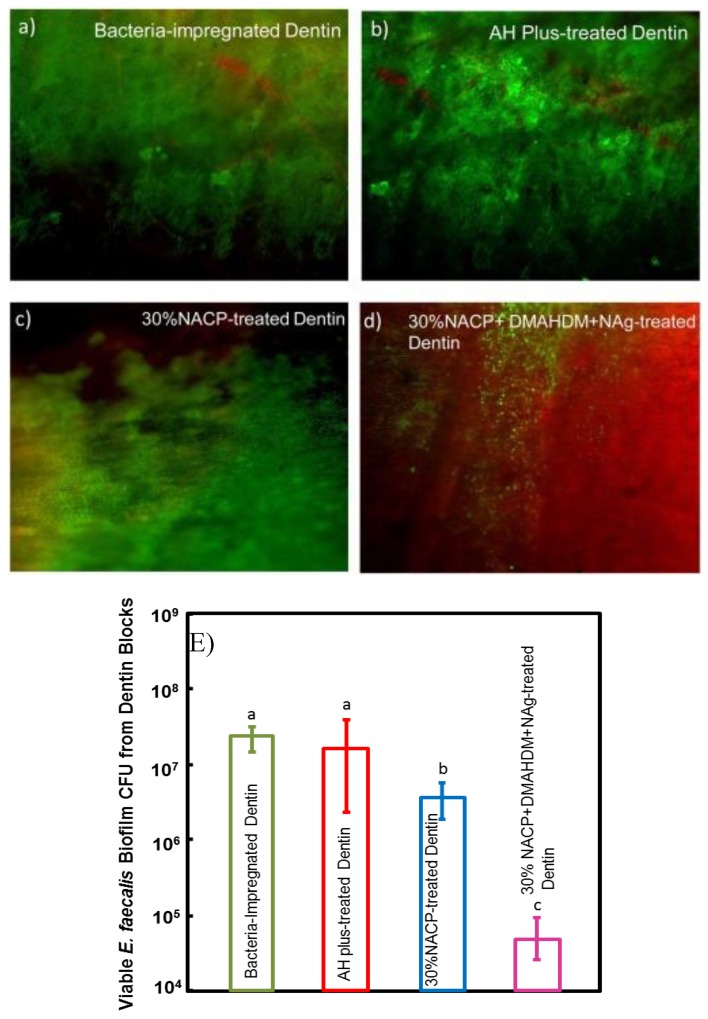
(**A**–**D**) Live/dead staining images of biofilms in dentin blocks. Live bacteria were stained green. Dead bacteria were stained red. Yellow/orange colors represent live and dead bacteria on top of each other. Dentin blocks of (**A**) bacteria-impregnated dentin control, (**B**) AH Plus-treated dentin, and (**C**) 30% NACP-treated dentin were mostly covered with live bacteria. Dentin blocks of (**D**) 30% NACP + DMAHDM + NAg were mostly covered with compromised bacteria. (**E**) Colony-forming unit (CFU) counts of bacteria impregnated in dentin Incorporating 5% DMAHDM + 0.15% NAg reduced the CFU counts by 3 orders of magnitude when compared to control groups. Adapted from [27] with permission.

Baras et al. combined NAg and DMAHDM into a root canal sealer and tested the effects of the dual incorporation of NAg and DMAHDM on *E. faecalis* biofilm inhibition [26]. Incorporating DMAHDM or NAg alone resulted in a 4 and 1 log reduction, respectively [26]. However, when both agents were combined, the CFU reduction was increased to 6 logs [26]. In another study, DMAHDM, NAg and NACP were combined into a root canal sealer and tested against biofilm inhibition using a dentin infection model, to account for possible inhibitory effects induced by dentin chemical composition [27]. When *E. faecalis*-impregnated dentin blocks were treated with sealers containing 5% DMAHDM and 0.15% NAg, biofilm, CFU of *E. faecalis*-impregnated dentin blocks was reduced by nearly 3 logs when compared to control groups and were mostly covered with compromised bacteria when viewed under an epifluorescence microscope following live/dead staining (Figure 5) [27]. 

In addition to the antibacterial properties imparted by the experimental sealer, the NACP-component of the sealer was responsible for the high release of Ca and P ions and increase in the dentin micro-hardness to match that of sound dentin [27]. Wang et al., combined DMAHDM and NACP with a protein repellant, 2-methacryloyloxyethyl phosphorylcholine (MPC), which is a methacrylate with phospholipid polar groups, that has previously shown to reduce protein adsorption and subsequent bacterial attachment [79].

They tested the triple incorporation of DMAHDM, NACP, and MPC against a 3-day and 14 day-mature multispecies biofilm composed of *Actinomyces naeslundii*, *Fusobacterium nucleatum*, and *E. faecalis* grown on saliva-coated sealer disks [79]. The mutual incorporation of 3% MPC and 5% DMAHDM together resulted in a CFU reduction of 3 logs, which was greater than that achieved by either agents alone [79]. 

## 7. Effects of Bioactive Additives on the Physical and Sealing Properties of Root Canal Sealers

One of the most important properties of root canal sealers is their ability to provide a hermetic seal at the sealer-dentin interface [23]. An optimal seal should prevent any leakage from the oral cavity or periapical tissues [22]. Leakage from oral fluids can, not only cause microbial contamination, but also degradation of the polymeric sealer material through hydrolytic plasticization [22]. Sealing properties of root canal sealers are usually assessed through bond strength testing or microleakage assessments [82]. The latter may be more relevant to endodontic practices as root canal sealers such as, AH Plus have previously demonstrated low bond strength measurements but performed well under microleakage assessments [82]. 

The effects of adding MDPB into root canal sealing systems on the bonding and sealing properties were previously investigated [57]. Results showed that when 5% MDPB was incorporated, the microtensile bond strength of the sealer system was significantly increased when compared to Epiphany sealer group [57]. When observed under scanning electron microscopy, The MDPB sealer system showed an intimate contact with root dentin without formation of any gaps [57]. Utilizing a fluid filtration test, the fluid flow rate for MDPB-experimental sealer after 1 week was zero [57]. After 4 weeks, the values for the MDPB sealer increased but were significantly lower than the Epiphany sealer group [57]. In another study, 5% DMAHDM, 3%MPC and 20%NACP incorporation into a root canal sealer system did not compromise the push-out bond strength and showed bond strength values that were similar to that of commercial control [79]. 

The microleakage potential of adding 5% DMAHDM and 20% NACP into a root canal sealer was also previously assessed utilizing a methylene blue dye penetration method [25]. The authors reported no significant difference in the rate of dye penetration rate when compared to AH Plus commercial control. SEM images revealed the ability of the NACP and DMAHDM containing sealer to infiltrate the dentinal tubules and create numerous resin tags [25]. Similarly, when 5% DMAHDM was combined with 0.15% NAg into an experimental root canal sealer, the sealing properties assessed through dye penetration were similar to that of AH Plus commercial control [26]. Another study showed no significant difference in the depth of dye penetration between EndoREZ commercial control and the sealers containing 1.25% and 2.5% DMADDM [61]. 

One explanation of the improved or maintained sealing and bonding properties of QAMs containing root canal sealers could be through their ability to inhibit host-derived and bacteria-derived matrix metalloproteinases (MMPs), which are known to accelerate the degradation of the sealer dentin bond [83,84,85]. Previous studies have demonstrated the ability of MDPB to inhibit soluble MMPs and matrix-bound dentin MMPs [83]. Indeed, another study showed the ability of DMADDM to achieve 90% inhibition of MMPs [84]. 

Another important property of root canal sealers is their ability to flow along the root canal and reach areas that remain untouched though instrumentation and irrigation techniques. According to the ISO specification 6876/2012, root canal sealers should have a minimum flow of 20 mm. When 5% DMAHDM and 20% NACP were combined with glass-filler mass fractions higher than 40%, the flow of the sealer was significantly reduced to values lower than that required by the ISO specifications [62]. However, sealer containing 40% glass-filler, 5% DMAHDM and 20% NACP showed a flow rate of (28.99 ± 0.69 mm) which meets the ISO requirements [62]. Similarly, when 0.15% NAg and 5% DMAHDM were combined into a root canal sealer, the flow rate of the sealer was similar to that of AH Plus commercial control and in accordance with ISO specifications [26]. In another study, the authors reported adequate flow properties when nanoparticles of QPEI were incorporated into RCS sealer [66]. 

Film thickness properties of root canal sealers are also very important, since root canal sealers are considered a weak component that is more susceptible to polymeric degradation in root canal filling systems. For that reason, root canal sealers are applied in thin films and most of the canal space is occupied by core filling material. When sealers were tested for their film thickness properties, the incorporation of 5% of DMAHDM and 0.15% of NAg into an experimental methacrylate resin root canal sealer did not influence the film thickness properties of the modified sealer and showed film thickness values that were similar to that of AH Plus commercial control [26]. The incorporation of triple agents of 5% DMAHDM, 0.15% NAg, and 30% NACP did not negatively influence the film thickness properties when compared to AH Plus control and were in accordance with ISO requirements 6278/2012 [27]. When Ag_3_PO_4_ particles were incorporated into the polyurethane root canal sealer at 1% and 3%, the film thickness properties of the developed sealer were not compromised and were in accordance with ISO specifications [43]. Although the incorporations of 10% of HAp, α-TCP, or OCP particles into an experimental methacrylate resin-based endodontic sealer produced flow values statistically lower than that of control groups, the film thickness values were not statistically different between the groups [32]. 

The solubility of root canal sealers could also determine the success of endodontic therapy, as sealers with low solubility could enhance the longevity of root canal treatment and prevent the creation of any passageways for bacterial leakage. When Seung et al. assessed the effects of adding 2.5% DMAHDM and 0.15% NAg into AH Plus, the solubility of the sealer was not compromised and showed solubility rates similar to that of unmodified AH Plus [24]. Similarly, the incorporation of 5% Ag_3_PO_4_ into a polyurethane root canal sealer showed solubility values that were in accordance with the requirements of the ISO specifications [43]. When DMADDM (1.25%, 2.5%) was incorporated into EndoRez sealer, no difference in the solubility between sealers with or without DMADDM was observed [61]. QPEI nanoparticles incorporated into RCS sealer also did not influence the solubility of the modified root canal sealer when compared to the unmodified sealer based on the ISO specifications [66]. 

## 8. Cytotoxicity of Antibacterial and Remineralizaing Additives

Ensuring that bioactive additives are compatible with mammalian cells is of paramount importance to allow their placement inside human teeth. Various studies have assessed the biocompatibility of many of these antibacterial and remineralizing additives. Although in those previous studies the bioactive additives were incorporated into methacrylate resin-based bonding agents and composite materials, similar results are expected if the same additives are incorporated into methacrylate resin-based root canal sealers.

In a previous study, the cytotoxicity of DMADDM (1.25%, 2.5%) incorporated into EndoREZ sealer was evaluated against mouse fibroblasts (L929) [61]. The results of the cytotoxicity test have shown that adding DMADDM at a mass fraction of 1.25% or 2.5% did not increase the cytotoxicity of the sealer when compared with unmodified EndoREZ [61]. When QPEI nanoparticles were incorporated into RCS epoxy-based sealer, it was found to be non-cytotoxic [66]. The QPEI endodontic sealer showed a decolorization zone of 0 and median cell lysis of 0 (0/0), interpreted as non-cytotoxic. 

The biocompatibility of MDPB was previously assessed against human pulpal cells [86,87]. MDPB showed low levels of cytotoxicity that were similar to that of dimethacrylate monomer, TEGDMA, a commonly used monomer in dentistry [87]. When the biocompatibility of MDBP was compared to Bis-GMA, against odontoblast-like MDPC-23 rodent-derived cells, the authors reported lower cytotoxic effects than those produced by Bis-GMA [88]. 

In a study conducted on rats, DMADDM and NACP were incorporated into a composite and adhesive system that was used to restore occlusal cavities prepared on first molars of rats [60]. The pulpal inflammatory response and tertiary dentin formation were all assessed [60]. Groups containing DMADDM+NACP showed better biocompatibility and less tissue disorganization than control groups [60]. At 30 days, restorations containing NACP demonstrated tertiary dentin formation four- to six-folds greater than that of the control [60]. 

When QAMs with varying alkyl chains were tested for their cytotoxicity against human gingival fibroblasts and odontoblast-like MDPC-23 cells, it was shown that increasing cytotoxicity was observed with increasing QAMs concentrations and longer alkyl chains [50]. However, all tested QAMs, including DMAHDM showed biocompatibility better than Bis-GMA and similar to that of TEGDMA and 2-hydroxyethyl methacrylate (HEMA) [50]. 

The cytotoxicity of silver in various compositions has also been previously assessed. The cytotoxicity of NAg has previously shown to be concentration dependent [38,89,90]. Concentrations up to 0.7% have previously shown favorable biological response against human cells [38,91]. To date, concentrations higher than 0.15% NAg have rarely been used in polymeric dental resins [38,92,93,94]. Although studies on the cytotoxicity of the aforementioned bioactive agents have shown favorable results, root canal sealers used as carriers for these additives have previously shown varying degrees of cytotoxicity [95,96]. Previous studies have shown that most root canal sealers from different classes show some cytotoxic effects upon their application [95]. While most commercial root canals sealers exhibit some degrees of cytotoxicity, healing of the periapical lesions and repair of the damaged periapical tissues still occurred [95]. To date, there has been no definitive conclusion on which type of root canal sealer presents the best biocompatibility.

## 9. Summary

The inclusion of novel, biocompatible and bioactive agents into dental products to achieve strong antibacterial and tissue-remineralization functions is highly desirable. These strategies are highly promising to improve the endodontic treatment outcomes and can potentially prevent and control endodontic diseases. However, methodologies that are more clinically relevant need to be employed in future studies to better assess the potential applicability of these new materials. In addition, materials with antimicrobial and remineralizing properties need to be assessed under in vivo conditions that better simulate the oral environment.

This article represents the first review to focus on the effects of the new generation of bioactive endodontic sealers with incorporation of bioactive additives on the antibacterial and tooth mineral-regeneration capabilities. Recent studies developed novel bioactive and therapeutic root canal sealers with antimicrobial properties through the incorporation of additives including QAMs and NAg, which were able to reduce biofilm CFU by 6 logs. In addition, nanostructured remineralizing fillers, such as NACP, have been used to strengthen the root structure and prevent future tooth fractures. Root canal sealers containing NACP were able to increase dentin hardness significantly to match that of sound dentin. QAMs, NAg and NACP showed minimal negative effects on physical and sealing properties and were shown to be non-cytotoxic to human cells. Therefore, these new therapeutic and bioactive materials have the potential of enhancing the endodontic treatment efficacy, protecting the tooth roots, and increasing the longevity of the tooth.

## Figures and Tables

**Table 1:** A summary of antimicrobial additives that have been incorporated into root canal sealers.

Antimicrobial Agent	Formulation	Concentration	Methodology	Major Findings	Ref.
Silver	Silver phosphate (Ag_3_PO_4_)	1 wt %, 3 wt %, and 5 wt %	Anti-adhesion assay and a direct contact antibacterial test (DCT) against *S. mutans*	Ag_3_PO_4_ at 3% and 5% demonstrated stronger antimicrobial effects than that achieved with 1% Ag_3_PO_4_ and an epoxy-resin sealer AH Plus	[43]
Silver	Nanostructured silver vanadate decorated with silver nanoparticles (AgVO_3_)	2.5 wt %, 5 wt %, and 10 wt %	Minimum inhibitory concentration (MIC) and agar diffusion test after 48 h and 7 days against *E. faecalis, P. aeruginosa,* and *E. coli.*	AgVO_3_ incorporated at all 3 concentrations into AH Plus showed no significant effect on the inhibition zones against *E. faecalis,* regardless of the incubation time. Sealer 26 with AgVO_3_ at 5% and 10% enhanced the antibacterial potency.	[44]
Silver and Quaternary ammonium compound (QAC)	Nanoparticles of silver (NAg), and dimethylaminododecyl methacrylate (DMAHDM)	NAg: 0.05, 0.1. and 0.15 wt %. DMAHDM: 2.5, and 5 wt %	A modified direct contact test (MDCT) evaluated antibacterial properties after days 1, 7, and 14 against *E. faecalis*	On day 1, AH Plus sealers with 0.15% NAg or 2.5% DMAHDM alone or in combination were significantly more effective against *E. faecalis* compared with AH Plus. On days 7 through 14, 2.5% DMAHDM sealer + 0.15% NAg combined with 2.5% DMAHDM continued to be significantly more antibacterial than unmodified AH Plus.	[24]
Silver and Quaternary ammonium compound (QAC)	Nanoparticles of silver (NAg), and dimethylaminododecyl methacrylate (DMAHDM)	NAg: 0.05, 0.1. and 0.15 wt % DMAHDM: 2.5, and 5 wt %	Colony forming units (CFU), Polysaccharide production, and live/dead analysis against *E. faecalis* biofilm.	Sealer with 0.15% NAg achieved 2 log CFU reduction. Combining 0.15% NAg and 5% DMAHDM resulted in 6 log CFU reduction	[26]
Chlorohexidine and Quaternary ammonium compound (QAC)	Chlorhexidine digluconate (CHX) and Cetrimide (CTR)	CHX: 1 and 2 wt % CTR: 0.1–0.5 wt %	DCT, Biofilm test: Calgary Biofilm Device (MBEC-high throughput [HTP]; Innovotech Edmonton, AB, Canada)	2% CHX incorporated into AH Plus sealer achieved 1.3 log CFU reduction. 0.5% CTR achieved 2.92 log CFU reduction. Combining 2% CHX with 0.5% CTR increased CFU reduction to 6.28	
Quaternary ammonium compound (QAC)	12-methacryloyloxydodecylpyridinium bromide (MDPB)	5 wt %	DCT against planktonic or adherent *E. faecalis* bacteria for 30 or 60 s. A root canal infection model studying antibacterial effects of MDPB against *E. faecalis* within dentinal tubules.	For Planktonic bacteria, MDPB achieved after 30 s contact, a 99.9% killing of planktonic bacteria and 100% killing was achieved after 60 s. For adherent bacteria, MDPB killed more than 98% in 30 s and 99% in 60 s. A 99.5% killing was achieved by MDPB for bacteria impregnated inside dentinal tubules.	[57]
Quaternary ammonium compound	Dimethylaminododecyl methacrylate (DMADDM)	0, 1.25, 2.5, and 5 wt %	DCT, CFU counts, crystal violet assay, scanning electronic microscopy (SEM) and live/dead analysis against multispecies bacteria (*E. faecalis*, *S. gordonii*, *A. naeslundii*, and *L. acidophilus*), in planktonic cells or biofilms. Fluorescence in situ hybridization and quantitative real-time polymerase chain reaction.	Sealer containing 2.5% DMADDM achieved a CFU reduction of 1 log and significant biofilm biomass reduction. SEM analysis showed the addition of DMADDM resulted in a looser biofilm structure. A significant reduction associated with the addition of DMADDM in the proportion of *E. faecalis.*	[61]
Quaternary ammonium compound	Dimethylaminododecyl methacrylate (DMAHDM)	5 wt %	DCT, CFU counts, polysaccharide production, live/dead analysis against *E. faecalis* biofilm.	5% DMAHDM achieved a 4 log CFU reduction and significant reduction in biofilm polysaccharide production by *E. faecalis*. Live/dead analysis revealed, sealers containing 5% DMAHDM were primarily covered with dead bacteria	[62]
Quaternary ammonium compound	Quaternary ammonium polyethyleneimine nanoparticles (QPEI)	1.5 wt %	DCT, ADT, CFU counts and SEM analysis against *E. faecalis.*	QPEI nanoparticles reduced number of viable bacteria by 6 logs. SEM analysis revealed sealer with QPEI demonstrated significant changes in bacterial morphology and distribution. Syncytium-like cells and bacterial lysis were observed on sealers containing QPEI nanoparticles	[66]
Chitosan	Chitosan nanoparticles (CNps)	1 g/150 mg	DCT, membrane-restricted antibacterial experiments, CFU counts against *E. faecalis*. Antibiofilm properties of CNPs sealer assessed using a root canal infection model and CLSM after dentin pretreatment with carboxymethyl-chitosan (CMCS) alone or combined with rose bengal.	Significant improvement in the antibacterial properties of sealer against *E. faecalis* after the incorporation of CNps. Sealers containing CNps showed significantly low viable biofilm total values and much less bacterial colonization.	[67]
